# Work experiences of ethnic minority nurses: a qualitative study

**DOI:** 10.1186/s13584-016-0076-5

**Published:** 2016-07-20

**Authors:** Yael Keshet, Ariela Popper-Giveon

**Affiliations:** Western Galilee Academic College, Acre, Israel; David Yellin Academic College, Jerusalem, Israel

**Keywords:** Arabs, Ethnic Groups, Israel, Sociological Factors, Minority Groups, Nursing

## Abstract

**Background:**

Recruitment and retention of a diverse ethnic workforce in healthcare settings contribute to the provision of culturally competent care in multicultural contexts. Nevertheless, the work experiences of ethnic minority nurses, which impact the attractiveness of the occupation, job burnout and turnover intentions, are not well understood.

The present exploratory research seeks to examine the work experiences of ethnic minority Arab nurses in Israeli public hospitals. Israel is an interesting case study as the number of Arab nurses operating in the Israeli workforce has risen significantly over recent decades; many of them work in mixed Jewish-Arab environments, which are affected by the Israeli-Palestinian conflict.

**Methods:**

In-depth interviews with 13 Arab nurses working in Israeli public hospitals.

**Results:**

The interviewed Arab nurses mentioned various benefits associated with the nursing profession, as well as various difficulties they encounter during their daily work, which are specific to them as ethnic minority nurses. They describe nursing as an occupation that offers numerous employment opportunities, job security, professional development and promotion. They believe that their work as a nurse contributes to the health of the Arab family and community and enhances culturally competent healthcare in Israeli hospitals. However, Arab nurses also feel they are stereotyped; they face disapproving looks, refusal to be treated by them, and incidences of hostility toward them. The dual experience of both integration and rejection shapes their coping strategies.

**Conclusions:**

The findings can inform a more systematic study that could potentially examine both nurses’ and patients’ conceptions of multicultural care. Action should be taken to ensure optimal working conditions for Arab healthcare professionals. Institutional policies and actions are needed to cope with their unique difficulties, such as the appointment of a functionary responsible for minimizing and coping with stereotypical and hostile attitudes.

## Background

Multicultural populations present health organizations, specifically those in the Western world, with the challenge of delivering care to patients with diverse healthcare beliefs, languages and practices. A culturally diverse healthcare workforce, which meets the needs of these increasingly diverse populations, is crucial to the effort to provide culturally competent and patient-centered care, improve access to high-quality healthcare services, refine management of the healthcare system, provide opportunities for the acquisition of culturally competent skills by all staff members, and help reduce health disparities [1]. A multicultural nursing workforce is particularly important since nurses make up the largest group of frontline healthcare providers, who are in close contact with patients from diverse cultural backgrounds, aiming to provide them with patient-centered care [2].

Whereas current efforts to guarantee an ethnically diverse nursing workforce have focused primarily on addressing the recruitment and retention of ethnic minority students [3–6], the job experiences of ethnic minority nurses are not well documented or understood. Xue [2] found that differences in nurses’ job satisfaction were observed across racial and ethnic groups, and called for further research into the factors underlying these differences, so as to enable nursing and hospital administrators to develop effective strategies toward improving job satisfaction and retaining ethnic minority nurses, steps that may increase ethnic diversity in the public healthcare system’s workforce.

Studying the benefits that the nursing profession offers ethnic minority nurses, on the one hand, and the difficulties they encounter during their ongoing work, on the other hand, may contribute to the understanding of the unique job experiences of ethnic minority nurses. This can be done by listening to what they have to say, paying attention to their particular experiences and assessing the unique benefits they gain and the difficulties they face in healthcare organizations. Exploring these experiences may help recruit and retain more ethnic minority nurses in the public healthcare system, enhance cultural competency and reduce health inequalities.

Qualitative research is an appropriate tool for exploring work experiences of nurses and this method has been widely used in a variety of contexts. For example: semi-structured interviews were conducted with nurses to explore their experiences of caring for palliative and terminal patients [7]; reflective diaries were used to capture the work experiences of directors of nursing clinics in a study conducted in South Africa [8]; and face-to-face semi-structured interviews were conducted to study the experiences of male nursing students in Hong Kong [9].

We used in depth-interviews to study the experiences of Arab nurses who work in Israeli public hospitals that serve a mixed Jewish-Arab population. Israel is an interesting research site. First, in Israel's healthcare system, both Jews and Arabs are employed as professionals and are treated as patients. Second, their interaction within the Israeli healthcare system is embedded in the unique context of the Israeli-Palestinian conflict. Studying Arab nurses' experiences in Israeli public hospitals can therefore shed light on the perceptions of ethnic minority nurses in general, and of those operating within conflictual contexts in particular.

### Arab nurses in Israel

The Arab minority is the largest ethnic minority in Israel, comprising about 20 % of the population [10]. Arabs differ from the Jewish majority in Israel in religion, culture and language. Although they enjoy full citizenship, the Arab population’s socioeconomic status is lower, unemployment rates are higher, education levels are lower and income is lower [11, 12]. Moreover, the ongoing national conflict in the region and the inferior political status of Arabs in Israel place tremendous pressures on this population [13]. Health disparities between Arabs and Jews persist. Mortality and morbidity rates among the Arab population are higher than among the Jewish population and life expectancy is lower [14].

Health disparities persist despite the fact that, since 1995, equality in the provision and quality of healthcare for all citizens, including the Arab citizens, is mandated by Israel’s National Health Law. In addition, Israel’s Ministry of Health requires health organizations to invest in the accessibility of health care services to populations of different cultural origin and recommends, whenever possible, to recruit medical, paramedical, and administrative personnel from various linguistic and cultural groups [15].

The Israeli healthcare system therefore provides relatively equal employment opportunities to Arab citizens [16, 17]. This stands in contrast to certain other fields of employment, such as the military industry and other security-related fields, which are practically barred to Arabs [18, 19]. Besides the conflictual political situation, another obstacle to Arabs’ successful integration into the Israeli labor market is their lower level of education. Despite a sharp rise in the level of education among the Arab population (particularly among Christian-Arabs) [20], the rate of academics among the Arab minority is lower than that of academics among the Jewish majority [17].

In light of the obstacles Arabs face in the Israeli labor market, the nursing profession constitutes an outstanding career path for educated Arabs. Birenbaum-Carmeli [21] pointed to the increasing numbers of Arab nursing students in Israeli institutions of higher education. As a result, the Arab population is well represented in the profession of nursing, and its nursing employment rates are similar to the proportion of Arabs in the general population. Data gathered during the years 2011–2013 reveals that while the relevant Arab population (aged 15 and over) constitutes 18.3 % of the total population, the proportion of Arabs among all Israeli employees is only about 12.8 %. Yet Arabs account for 18.4 % of all nurses [22]. Nursing is a common employment path not only for Arab women [23] but also for Arab men [24, 25]. Even though nursing is a traditionally female occupation, nursing constitutes a major employment path for Arab men in Israel, who choose this profession far more frequently than do Jewish men. A total of 38.6 % of all Arab nurses were men, compared to only 7.5 % of Jews [25].

Scholars who examined what motivated Arab students to take up nursing found that they mentioned altruism, a desire to help others, followed by professional interest [26]; as well as the therapeutic aspect of the profession, professional practices (such as authority and responsibility) and employment conditions (such as working hours and wages) [27]. In their qualitative study, Arieli and Hirschfeld [28] found that while among Jewish nursing students pragmatic considerations constituted a relatively minor motivation for choosing nursing, almost all the Arab students they studied reported that their choice of nursing was basically rooted in pragmatic and instrumental considerations, since the profession would assure them relatively secure and gainful employment. Popper-Giveon and Keshet [25] likewise showed that Arab male nurses chose to practice nursing primarily because of the economic benefits of the profession, which enable them to perform the social role expected of young Arab men in Israel, namely to acquire property, build a home, marry and start a family.

Most of these studies focused on the Arab students' motivations to choose the nursing profession. Their findings are important because they may help to recruit Arab nurses to the Israeli healthcare system. One particular research project [28] examined the ways Arab and Jewish nursing students, who study side by side in an academic school of nursing located in northern Israel, perceive each other, as well as the nature of the relationships among them. This study, which was based on semi-structured interviews, revealed that the students perceive themselves as constituting two separate groups according to nationality. While cooperation between the groups, which focuses mainly on study-related tasks, is described as generally satisfactory, social distance across these two groups is clearly felt. Another qualitative study of Arab nurses in Israel [29] addresses the experiences of registered nurses during their studies toward their Bachelor’s degree. Focusing on minority-majority relations in mixed work Jewish-Arab teams in healthcare organizations, these studies found that nurses cope quite effectively in their daily practice. However, working in a mixed team does tend to arouse anger, animosity, disrespect, tension and alienation, which, nurses reported, impede their functioning at work.

The findings indicated the presence of implicit discrimination and tensions associated with the salience of social categorization. These tensions exacerbated in crisis situations. In calm periods nurses manage to draw on similarities and embrace shared identities. Their major coping pattern was to deflect their disagreements to a “hidden sphere”. These nurses are largely left to their own devices in coping with these deep social and national schisms. These studies focused on working relations in mixed teams and cooperation among their members.

In line with these previous findings, the purpose of our research was to explore work experiences of Arab nurses in Israel, with a view to using our findings to plan further research on the subject of ethnic minority nurses in a multicultural society. We focused on the benefits gained and difficulties experienced by Arab nurses in their ongoing work in Israeli public hospitals, which serve both Jewish and Arab patients and employ both Jewish and Arab professionals. We emphasize mainly the experiences that are specific to them as members of an ethnic minority population; experiences that may contribute to our understanding of ethnic minority nurses in other multicultural contexts as well, and may have implications on job satisfaction, recruitment and retention of ethnic minority nurses in healthcare organizations.

## Methods

During 2014 we conducted 13 face-to-face semi-structured, in-depth interviews with a convenience sample of Arab nurses working in Israeli public hospitals. Participants were recruited using snowball sampling. We chose to focus on Arab nurses employed in public hospitals in mixed cities (such as Jerusalem and Haifa), who treat both Arab and Jewish patients, positing that this unique setting could better contribute to our understanding of the multicultural context in which Arab nurses in Israel practice. We interviewed seven male nurses and six female nurses, ranging in age from 23 to 60 (mean age 31). All interviewees were Muslims, apart from one Christian, a ratio reflecting that of Israel’s overall Arab population. All nurses graduated in Israel, either in prestigious universities or in local colleges. The participating nurses were employed in an internal medicine ward (1), a delivery room (1), a women’s medicine ward (2), a children’s ward (1), a surgical ward (1), a children’s surgical ward (1) and in an emergency room (6). We sought to locate participants from a variety of work environments in order to widen the scope of the study and encompass multiple voices, opinions and experiences that could yield a preliminary map of the different dimensions in the work experiences of Arab nurses in Israel.

Interviews lasted between 45 and 90 min. We conducted one interview with each participant, in order to draw on the different dimensions of their work experiences. We therefore asked the Arab nurses about their unique experiences as members of an ethnic minority group within the predominantly Jewish Israeli healthcare system; and about their decision to study nursing, their work experiences in Israeli healthcare organizations, and their interactions with Jewish and Arab patients and colleagues. Interviews were conducted in Hebrew, in which all researchers and participants were fluent.

Interviews were recorded, transcribed verbatim and analyzed by the two authors in order to map key issues, such as the interviewee's childhood dream, the motivations to choose nursing, and work experiences. The authors read through the transcripts to assemble a set of codes, according to which the data structure was organized. The codes were then arranged according to higher level categories (such as rewards and hardships encountered) and analyzed to identify relationships among them. The quotes, spoken in Hebrew, were translated into English while adhering to their original spirit.

This research brings together researchers from the Jewish majority and interviewees from the Arab minority in Israel. This meeting is charged, especially since the interviews were conducted in the context of violent incidents and national and religious conflicts. These circumstances furthermore reflect the gap in power relations that exists in Israeli society, including its health system, and exemplify the dual experiences of Arab nurses in Jewish-dominated organizations: the desire to be appreciated and to integrate on the one hand, while feeling distance and alienation on the other. We feel that the national and religious tension on the “outside,” alongside the encounter with researchers from the majority population, induced the interviewees to refer to issues of social integration and racism.

The ethics committee of the Western Galilee College approved the study. All the participants were given information on the study; they agreed to participate on a voluntary basis. No incentive was offered, and the anonymity of the participants was preserved. All the names in the manuscript are pseudonyms.

### Findings

Our interviews with the ethnic minority Arab nurses in Israel revealed both the benefits and the difficulties these nurses experience during their daily work. The nurses talked about benefits and difficulties that are relevant to nurses in general, as well as those that are relevant and meaningful specifically to them as ethnic minority nurses. In accordance with the purpose of this article, we concentrate on the latter.

### Perceived benefits

The interviewed Arab nurses mentioned various benefits of the nursing profession that are particularly relevant to them as ethnic minority nurses. These included relatively accessible employment opportunities, job security and promotion possibilities; an opportunity to improve the status of Arab women; a way to contribute to the health of the Arab family and community and a way to enhance the culturally competent healthcare offered in Israeli hospitals.

#### “You can always find work”: Employment opportunities

A prominent benefit that the occupation of nursing offers Arabs in Israel is fairly rapid and smooth integration within the Israeli labor market. The Israeli healthcare system provides equal employment opportunities to Arab academics, who generally encounter major obstacles when seeking to enter the Israeli labor market [18, 19]. Fares (a male nurse) points to this benefit, when comparing nursing to the profession of electric engineering:

Today, the paramedical occupations… such as nursing… are almost the only working option for the Arab sector… There are many occupations in which Arabs cannot integrate. The paramedical occupations are the most appropriate and the market is open… Let’s say, if someone studied electrical engineering, he cannot easily find a job in the electricity company… compared to one who studied nursing, and can easily find work in any hospital… It has to do with demand (Fares).

Practicing nursing does not only allow employees from the Arab minority to join the Jewish dominated Israeli workforce, but also to enjoy job security, a protective labor union (the Israeli Association of Nurses is one of the strongest unions in Israel), and generous social benefits. The interviewees, some of whom have worked in the profession of nursing for many years, see it as providing fair and stable wages and offering relatively good working conditions, especially for minority employees.

(Nursing) promises security and a good salary too. There is also a shortage of nurses… wherever you go, you can always find work. I even thought about the option of working abroad. Nurses enjoy job security also abroad, they can also develop professionally. And I, I intend, after I marry my fiancé… he is also a nurse, we met at the university… so we will go to work, maybe in Canada or Australia (Zainab).

#### “It is not so common for women to leave [home] at night for work… of course they talk about me, but I don't care”: Improving the status of Arab women

Many Arab women in Israel, particularly in mixed cities such as Jaffa and Haifa, are exposed to processes of modernization and westernization. They are turning to higher education in growing numbers and integrate in the labor market. However, to some extent they remain subject to traditional gender imperatives, and are expected to fulfill, first and foremost, the roles of wife and mother. Practicing nursing allows educated Arab women to develop professionally, to pursue higher education, gain freedom of movement and establish contact with strangers. Nursing allows Arab women to work in Jewish population centers, even during night shifts, and to hold training and management positions involving responsibility and authority. Since it is associated with feminine and motherly traits such as caring and nurturing, nursing is considered a legitimate occupation for women. Consequently, it allows Arab women to develop professionally without violating the traditional gender norms.

There are things that are not accepted for women to do… I come from a small and quite religious village… where all are Muslims… of course they talk about me, but I don't care. I care only about my family. Perhaps it is not so acceptable that a young woman leaves home to work in the middle of the night, or returns at night from work … it is not because of nursing, but because of the night shifts (Zarifa).

#### “Nurses are good for the family”: Contribution to the health of the Arab family and community

The interviewed nurses explained that the medical knowledge they have acquired serves as a resource for the Arab family and community as a whole. This available and accessible medical knowledge is important, particularly since many of the Arab communities are located in peripheral locations in the north and south of the country that have less access to available services [14]. Fatma, for example, indicates that an Arab nurse is a resource in the community, especially among communities in rural or unrecognized villages, which lack many infrastructures and services. In such communities, those who possess medical knowledge are greatly appreciated.

The family actually encouraged me to study nursing. You know, a medical professional at home is always appreciated and the parents will always want someone to take care for them… I think every mother wants a nurse at home… It gives a lot. Health – there is nothing more important than health. Money comes and goes; work comes and goes, but health… When there is someone to help you as you get older – it is important (Farouk).

Arab nurses can contribute most within community healthcare services in which the professionals are predominantly Jewish, and the communication that takes place between them and Arab applicants is far from satisfactory. In these cases, Arab nurses can make a substantial contribution to health promotion in the Arab community in Israel, which enjoys fewer resources:

They contribute to the Arab sector. There are many Arab patients who do not speak Hebrew, who do not understand… For example, Arab nurses give guidance at baby clinics. In X (an Arab neighborhood in Jerusalem), there are two Jewish nurses and one Arab nurse, and all the Arab women go to the Arab nurse. They receive explanations and guidance, about the side effects of vaccines, drugs, what to eat and what not to eat… so it contributes to the promotion of health and the proper growth and development of children (Ahlam).

#### “I know the Arab society, I know how to approach them, how to talk to them, how they think. So it improves the care, the quality of care”: Contribution to culturally competent healthcare

Participants indicate that treatment by Arab nurses can benefit Arab patients, especially when the physicians are Jews, most of whom do not speak Arabic. The participants feel that they can help Arab patients obtain better care because they are familiar with the language and values of the Arab society in Israel. In other words, the Arab nurses are aware of their role in enhancing culturally competent care and in reducing the health disparities that adversely impact the Arab minority in the Israeli healthcare system.

Some interviewees pointed to the significant place language has in the medical care that minority patients receive. Since Arab professionals and Arab patients speak (and understand) the same language, treatment is improved and becomes more precise.

First of all – communication; It is important to communicate with the patient. There are those who actually know no Hebrew, and it is important to communicate. If you do not communicate, you do not understand. You do not know how to handle the patient (Abir).

Beyond their mastery of the Arab language, Arab nurses can contribute to the cultural mediation required in public hospitals. Arab nurses can mediate the expectations of Arab patients, their values and perceptions to the Jewish staff members, especially to physicians.

Diversity is very important in this profession. And the more you know the population, their culture, their therapeutic approach, the better, that is my belief (Samir).

### Perceived difficulties

The interviewed Arab nurses pointed to the many difficulties associated with nursing work. Some of these apply to nurses in general irrespective of their ethnicity: stressful working environments, conflicts with colleagues and patients, excessive job demands, the physical effort involved in the work of caring; the emotional load following one’s encounter with suffering, misery and death; cumulative burnout; heavy professional responsibility; the hardships of working shifts (including night shifts); and the challenge of balancing family life and career.

But, apart from these difficulties that challenge nurses in general (e.g., [30, 31]), we heard from the interviewees about further difficulties, particular to ethnic minority nurses employed in health organizations that are dominated by the majority culture. In other words, Arab nurses in Israel are obliged to cope with additional difficulties that add tension and burnout to their routine work. Among these difficulties are stereotypical and offensive attitudes on the part of patients and their families, and tense working relationships with Jewish colleagues.

#### “When they hear my name, they immediately know that I am an Arab”: Being stereotyped

The interviewed Arab nurses feel that they are stereotypically perceived by the patients (who are mostly Jews) as “Arabs.”

At first, they do not know that I am an Arab. When they hear my name, they immediately know that I am an Arab, and then they go through all the stereotypes they have in mind. Once, I took care of a patient who spoke English. She is Jewish, but speaks English all the time, and I speak English too, really well, even with an American accent. So we started talking and she just told me, “but you look more like an Israeli, you do not look like an Arab.” I told her that I'm an Israeli, “so you mean I don’t look like a Jew? I look like an Arab?… Well, I am an Arab.” I asked her: “Do you think all Arabs wear headscarves, and are not intelligent or educated?” (Zainab).

Some of the interviewees talked about a suspicious attitude on the part of patients, about hostile looks and a patronizing approach:

The patients are very nice. Really, most of them… But you feel a certain group, the extremists, they look at you – Why are you here? Why are you treating me? How come you work in the emergency room? You can see that look in their eyes (Ataf).

Some of the Arab nurses described far more unpleasant encounters with Jewish patients, some of whom refuse to receive treatment from them, and even behave violently.

Sometimes yes, we encounter racism from patients, “Go to Gaza” and things like that (Muammar).

Many patients came and told me "You are an Arab, we do not want you to take care of us." Even patients’ relatives said this… Some people act aggressively, and start beating you and so forth. So it's hard. I come to help you, and you are attacking me? Someone even punched me once. He called me "dirty Arab, go away!" and punched me. What can you do? It's impossible to transplant a brain to people; I'm not going to educate them; this is the way they think. There are tensions in this country that make them think like this: Arab – Jewish (Samir).

Sometimes it is merely the fear that a patient will refuse treatment that bothers the nurses; the suspicion that hangs over treatment sessions involving a nurse who belongs to the minority group and a patient from the majority population:

When touching and tending to a mother in the delivery room, you have to deal with… [intimate] parts… So I would always ask myself: “How will they take to that?” You know, these things are really, really personal, so how can I – will they accept me or not? If they do not accept me, what should I do? And if they do accept me, what should I do?… [Samira]

The stereotypical views, hostile glances and refusal of treatment that the Arab nurses describe are impacted by the pervasive violence that characterizes the Jewish-Palestinian conflict in the Middle East. In mixed Jewish-Arab cities such as Jerusalem and Haifa the national conflict penetrates the hospital, although it is often perceived as an egalitarian, humanistic and protected space.

It may be that here, at the hottest place in Jerusalem, there is more racism. We see now all sorts of terror attacks, so people perhaps look at us a little differently. They look at the name [on the tag] and they check. It is not easy now, but what can you do? That's the reality; we have to live in it (Musa).

#### “When everything is fine out there, so you feel it less”: Uneasiness with colleagues

The difficulties faced by Arab nurses in Israeli public hospitals, which are dominated by Jews, are due not only to the hostile attitudes of patients and their relatives. Sometimes tensions arise among the mixed teams, in which Arabs and Jews work together under a stressful workload.

Many of the interviewees described their working relations with their Jewish counterparts in harmonious, even idyllic terms.

In [the name of the hospital], I actually see a very beautiful coexistence. I personally love to see it. Everyone works in one team, loving each other (Zarifa).

However, when the external violence associated with the national conflict permeates into the team relationships, even the sterile and protected space of the hospital becomes an arena of confrontation.

My team is nice, I'm fine, but if there is a terror attack nobody looks at me, as if I am weird… You know, with the recent events, as if I was not one of the team, I did not feel like coming here at all. So I was like, "Why? Why? I didn't do it and I'm good with you and I help everyone, so why to treat me like that?" (Samira).

Ataf, also employed at a large hospital in Jerusalem, wishes to move to the north of Israel, where much of the Arab population resides, because he feels unsafe in Jerusalem. He blames the bad relations in the ward, the stereotypes and the violent atmosphere of the city's political situation.

Once there is something out there, you feel a little more uneasy in terms of interpersonal interaction with those around you. When everything is fine out there, so you feel it less. Some people are frightened. People are on the defense and I can well understand why… But still, the fact that people can feel threatened, it ultimately reflects on you and you feel uncomfortable. So next week I'm going to submit my forms… I want to try to move up north, to Haifa. I believe it is a little more relaxed there… The situation here is getting harder each day (Ataf).

#### “I kind of just went along with it, what could I do?”: Coping with the experience of confusing duality

Our interviews with the Arab nurses, who work in public Israeli hospitals, revealed a complex situation. On the one hand, as we have seen, the Israeli public healthcare system offers relatively equal employment opportunities to Arab academics, which enables them to join the Israeli labor market. But on the other hand, within these equal spaces the interviewees experience rejection, stereotypical responses, racist attitudes and even aggression on the part of patients and their families, and at times feel tension and uneasiness while working with their Jewish colleagues. This duality lies at the core of our findings. Jamil, an Arab male nurse, reflected on the contrast between the medical ethos of equality and impartiality, according to which he works, and the attitudes of some patients:

I personally treat anyone, I don’t care who he is. He is a human being. He has the right to live, he deserves dignity. It makes no difference what he does outside [the hospital], whether he’s a criminal, a thief, or does all kinds of things. We are not judges, nor the police, we don’t punish people. I take care of him, no matter what. But you know, I worked at the clinic, and there I felt that some people may have refused to be treated by me because I’m an Arab (Jamil).

This duality is confusing. The Arab nurses working in Israeli hospitals experience both integration and rejection. The coping strategies they use stem from this duality. The Arab nurses prefer to understate these difficulties and refrain from speaking about them. They try to downplay unpleasant incidents in order to preserve the humane and universal space of the hospital (or perhaps only to convince themselves that this exists). Since they may hesitate to acknowledge that racism and discrimination are prevalent, in their responses they seek to dismiss the pain and the incidents that cause it.

Patients are very nice. Really, most of them… They come for treatment and go home. But you do feel that part of the population, the extremes, yes, they may look at you: hey you, what are you doing here? … You can see it in their eyes. But this is a very small part of the population. It’s not really something that should affect my work (Ataf).

When a patient refuses to be treated by them, the Arab nurses submit and hand the patient over to a Jewish nurse. They generally don’t speak about it and do not complain, but such incidents undermine their confidence and cloud their mood.

I don’t like it (that Jewish patients refuse my treatment because I’m an Arab); I don't think it’s acceptable. In such cases I do as little as possible… I don’t talk to them, I do what I have to or simply let someone else attend to them (Ahlam).

Informants also reported how they would withdraw into their shell when they feel insulted by a fellow nurse. Ahlam told us about an incident that occurred during the course of hostilities between Israeli forces and the Palestinians in Gaza:

Someone said: Let’s erase the Gaza strip and all the Arabs… I kind of just went along with it, what could I do? I could have asked the nurse in charge, “perhaps you can deal with her, perhaps you can help me.” But other than that I can do nothing. I will always have to work with her and that’s that (Ahlam).

## Results and conclusions

The Arab nurses we interviewed described the various benefits, that they believed the profession of nursing offers them as members of a minority group. These include relatively accessible employment opportunities, job security and promotion possibilities; an opportunity to improve the status of Arab women; a way to contribute to the health of the Arab family and community; and a means to enhance the culturally competent healthcare offered to Arab patients in Israeli public hospitals.

At the same time, interviewees described the difficulties they experience in their everyday work at public hospitals in Israel. They described stressful working environments, stiff job demands, and physical and emotional hardships that resemble those reported in other studies [30]. Lee and Yom [32], for example, describe nurses who suffered from work stress and compassion fatigue. These factors, along with age, physical/psychological symptoms, job satisfaction, work engagement and work environment, were found to predict burnout among Taiwanese nurses [33].

Beyond these general difficulties that many nurses encounter in their ongoing work, the interviewees, as members of an ethnic minority group employed in health organizations dominated by the majority society, are obliged to cope with additional hardships. Among the difficulties suffered by Arab minority nurses we found stereotypical and offensive attitudes on the part of patients and their families, and tense working relationships with Jewish colleagues.

Yet these difficulties represent an unspoken phenomenon. It is not articulated by public policy managers. The interviewed Arab nurses themselves prefer not to speak about it, perhaps as a result of their dual experience. This duality leads them to suppress the hardships associated with their status as members of an ethnic minority, to remain silent, to avoid conflict. Their stories about the distress they encounter through belonging to a minority are “silenced voices” [34, 35]. The qualitative method we used – in-depth face-to-face interviews – allowed us to reveal these silenced voices. Traditional research paradigms, texts and theories used to explain the experiences of members of ethnic minorities have been criticized for silencing and distorting their experiences [36]. A qualitative research method, on the other hand, which encourages intimate conversation and induces interviewees to reveal their inner feelings, enables us to recognize such silenced voices in the qualitative data [35, 36]. In-depth interviews allowed us to illuminate the complexities of this silenced and unspoken phenomenon.

Previous studies conducted in other societies have also found that ethnic minority nurses suffer greater hardship and stress than non-minority nurses. Deery and colleagues [37] examined the effects of harassment on job burnout and turnover among ethnic nurses in British hospitals. Results suggest that the incidence of harassment was higher among ethnic minority nurses than among white nurses. Shields and Wheatley [38] also found that nurses from ethnic minority backgrounds in the UK report significantly higher levels of harassment and aggression in their workplace [38]. Indeed, it has been stated that for many black and Asian staff in the NHS ‘harassment and bullying, from both colleagues and patients are daily facts of life’ [39:13]. Likewise, a study of members of the Royal College of Nursing found that 45 % of African and Caribbean nurses had been harassed or bullied by a member of staff, compared to 21 % of white British nurses [40].

Our study may have implications for other Arab health professionals, such as physicians and pharmacists, who work in public health organizations in Israel. It seems that physicians are less exposed to hostile attitudes on the part of patients than are nurses [41]. This can be explained by the nurses’ closer contact with patients, by their lower prestige and authority, and by their tendency to react more passively and with greater restraint to the rejection and aggression displayed by patients. Pharmacists from the Arab minority in Israel, who are employed in mixed (Jewish-Arab) health organizations, were also found to experience discrimination and racism [42]. Their mode of coping presents a unique pattern, since they are "protected" behind the counter and enjoy higher prestige.

The findings of this exploratory research may well inform a more systematic study that could examine both nurse and patient outcomes associated with multicultural care. Since academically trained Arabs employed in nursing derive significant benefits, they tend to find it difficult to leave the profession, despite the high levels of stress and the singular hardships they face as a minority in the Israeli public healthcare system. However, the need to cope with everyday difficulties may affect the quality of work of Arab nurses in Israel. Therefore, action must be taken to ensure optimal working conditions for them. Institutional measures, such as the appointment of a functionary responsible for minimizing and coping with stereotypical and hostile attitudes toward Arab nurses (and other health professionals), are needed.

Along with ensuring optimal working conditions for nurses, patients’ experiences and satisfaction are no less important. Quality of nursing has been found to be a major factor associated with patients’ experiences and satisfaction [43–46]. Employment of non-UK educated nurses in English NHS hospitals was found to be associated with lower patient satisfaction and to impact the quality of care [46]. A study designed to examine whether patient satisfaction with nursing care is associated with the proportion of Arab nurses providing care [such as in 46], might be conducted in Israel to systematically evaluate how the presence of Arab nurses impacts patients’ satisfaction in Israeli hospitals. Such research could inform policies pertaining to the nursing workforce in the country.

As an exploratory research project, which examines how Israel’s multicultural health system is reflected in interviews with Arab nurses, this study has several limitations. It is based on a very small sample of participants, all of whom are Arab health professionals. We cannot draw generalizations from this study. A more comprehensive and quantitative study is needed in order to verify our findings and to address patients’ points of view as well. Future research is also needed to assess the levels of stress and burnout among Arab minority nurses in comparison to these levels among nurses who belong to the majority Jewish population, and to examine ways to reduce burnout as far as possible. Such a study could provide findings relevant to policy making, and thereby contribute to the formulation of appropriate policies. Moreover, it can contribute to the development of theoretical insights regarding the impact of the work environment on burnout of minority nurses.

## References

[1] Xue Y, Brewer C (2014). Racial and ethnic diversity of the U.S. national nurse workforce 1988–2013. Policy Polit Nurs Pract.

[2] Xue Y (2015). Racial and ethnic minority nurses’ job satisfaction in the U.S. Int J Nurs Stud.

[3] Gardner J (2005). Barriers influencing the success of racial and ethnic minority students in nursing programs. J Transcult Nurs.

[4] Walker MCD (2007). Commentary on ‘The relationship between cultural competence education and increasing diversity in nursing schools and practice settings’. J Transcult Nurs.

[5] Abriam-Yago K, Yoder M, Kataoka-Yahiro M (1999). The Cummins model: a framework for teaching students for whom English is a second language. J Transcult Nurs.

[6] Villarruel A, Canales M, Torres S (2001). Bridges and barriers: educational mobility of Hispanic nurses. J Nurs Educ.

[7] Tunnah K, Jones A, Johnstone R (2012). Stress in hospice at home nurses: a qualitative study of their experiences of their work and wellbeing. Int J Palliat Nurs.

[8] Munyewende PO, Rispel LC (2014). Using diaries to explore the work experiences of primary health care nursing managers in two South African provinces. Glob Health Action.

[9] Chan ZC, Lui CW, Cheung KL, Hung KK, Yu KH, Kei SH (2013). Voices from a minority: experiences of Chinese male nursing students in clinical practice. Am J Mens Health.

[10] ICBS (Israel Central Bureau of Statistics). Table B/1. Population, by population group. 2015; http://www.cbs.gov.il/publications15/yarhon0815/pdf/b1.pdf. Accessed 17 Jan 2016.

[11] Herzog H (2004). ‘Both an Arab and a woman’: Gendered racialized experiences of female Palestinian citizens of Israel. Soc Identities.

[12] Baron-Epel O, Kaplan G (2009). Can subjective and objective socioeconomic status explain minority health disparities in Israel?. Soc Sci Med.

[13] Rouhana N (1998). Israel and its Arab citizens: predicaments in the relationship between ethnic states and ethnonational minorities. Third World Q.

[14] Averbuch E, Avni S (2014). Inequality in Health and Coping with it.

[15] Ministry of Health (2011). Providing Cultural and Linguistic Accessibility in the Health System. General Administration Circular.

[16] Popper-Giveon A, Liberman I, Keshet Y (2014). Workforce ethnic diversity and culturally competent health care: the case of Arab physicians in Israel. Ethn Health.

[17] Keshet Y, Popper-Giveon A, Liberman I (2015). Intersectionality and underrepresentation among health care workforce: the case of Arab physicians in Israel. Isr J Health Policy Res.

[18] Sharabi M (2014). The relative centrality of life domains among Jews and Arabs in Israel: the effect of culture, ethnicity and demographic variables. Community Work Fam.

[19] Yashiv E (2012). Israeli Arabs in the Labor Market.

[20] Yashiv E, Kassir N. The labor market of Arabs in Israel. Tel Aviv: Tel Aviv University [Hebrew.] 2013; file:///C:/Users/mom/Downloads/IsraeliArabs_policy_paper.pdf.

[21] Birenbaum-Carmeli D (2007). Contextualizing nurse education in Israel: Sociodemography, labor market dynamics and professional training. Contemp Nurse.

[22] Popper-Giveon A, Keshet Y, Liberman I (2015). Israeli Arabs in Health and Welfare Professions: an Ethnic and Gender Oriented View of Representation and Employment. Social Security J.

[23] Yonay Y, Kraus V. Ethnicity, gender and exclusion: Which occupations are open to Israeli Palestinian women? In: N. Khattab & S. Miaari (eds.), Palestinians in the Israeli labor market: A multi-disciplinary approach. 2013; London: Palgrave MacMillan (p. 88–110).

[24] Romem P, Anson O (2005). Israeli men in nursing: social and personal motives. J Nurs Manag.

[25] Popper-Giveon A, Keshet Y (2015). Increasing gender and ethnic diversity in the healthcare workforce: The case of Arab male nurses in Israel. Nurs Outlook.

[26] Halperin O, Mashiach-Eizenberg M (2014). Becoming a nurse — A study of career choice and professional adaptation among Israeli Jewish and Arab nursing students: A quantitative research study. Nurse Educ Today.

[27] Haron Y, Reicher S, Riba S (2014). Factors influencing nursing career choices and choice of study program. Health Mark Q.

[28] Arieli D, Hirschfeld MJ (2010). Teaching nursing in a situation of conflict: Encounters between Palestinian-Israeli and Jewish-Israeli nursing students. Int Nurs Rev.

[29] Desivilya H, Raz H (2015). Managing diversity and social divisions in nurses’ work teams. EuroMed J Business.

[30] Khamisa N, Oldenburg B, Peltzer K, Ilic D (2015). Work Related Stress, Burnout, Job Satisfaction and General Health of Nurses. Int J Environ Res Public Health.

[31] Chayu T, Kreitler S (2011). Burnout in Nephrology Nurses in Israel. Nephrol Nurs J.

[32] Lee UM, Yom YH (2013). Effects of work stress, compassion fatigue, and compassion satisfaction on burnout in clinical nurses. J Korean Acad Nurs.

[33] Lee HF, Yen M, Fetzer S, Chien TW (2015). Predictors of burnout among nurses in Taiwan. Community Ment Health J.

[34] Maple M, Edwards H, Plummer D, Minichiello V (2010). Silenced voices: hearing the stories of parents bereaved through the suicide death of a young adult child. Health Soc Care Community.

[35] Lincoln Y, McLaughlin D, Tierney W (1993). I and Thou: Method, voice, and roles in research with the silenced. Naming silenced lives.

[36] Solórzano DG, Yosso TJ (2002). Critical race methodology: counter-storytelling as an analytical framework for education research. Qual Inq.

[37] Deery S, Walsh J, Guest D (2011). Workplace aggression: the effects of harassment on job burnout and turnover intentions. Work Employ Soc.

[38] Shields MA, Wheatley PS (2002). The determinants of racial harassment at the workplace: evidence from the British nursing profession. British J Indust Relat.

[39] Iganski P, Mason D (2002). Ethnicity, Equality of Opportunity and the British National Health Service.

[40] Royal College of Nursing (2005). At Breaking Point? A Survey of the Wellbeing and Working Lives of Nurses in 2005.

[41] Keshet Y, Popper-Giveon A. Race-based experiences of ethnic minority health professionals. In process. 10.1080/13557858.2017.1280131 28100067

[42] Popper-Giveon A, Keshet Y. The white coat trap: Pharmacy as an ehnic-dominated occupation in Israel. In proccess.

[43] Kutney-Lee A, McHugh MD, Sloane DM (2009). Nursing: a key to patient satisfaction. Health Aff.

[44] Jha A, Orav E, Zheng J (2008). Patients’ perception of hospital care in the United States. N Engl J Med.

[45] Aiken LH, Sermeus W, Van den Heede K (2012). Patient safety, satisfaction, and quality of hospital care: cross sectional surveys of nurses and patients in 12 countries in Europe and the United States. BMJ.

[46] Germack HD, Griffiths P, Sloane DM (2015). Patient satisfaction and non UK educated nurses: a cross sectional observational study of English National Health Service Hospitals. BMJ Open.

